# A Large-scale Synthetic Pathological Dataset for Deep Learning-enabled Segmentation of Breast Cancer

**DOI:** 10.1038/s41597-023-02125-y

**Published:** 2023-04-21

**Authors:** Kexin Ding, Mu Zhou, He Wang, Olivier Gevaert, Dimitris Metaxas, Shaoting Zhang

**Affiliations:** 1 Department of Computer Science, University of North Carolina at Charlotte, Charlotte, NC 28262 USA; 2 Sensebrain Research, San Jose, CA 95131 USA; 3 Department of Pathology, Yale University, New Haven, CT 06520 USA; 4 Stanford Center for Biomedical Informatics Research, Department of Medicine and Biomedical Data Science, Stanford University, Stanford, CA 94305 USA; 5 Department of Computer Science, Rutgers University, New Brunswick, NJ 08901 USA; 6 Shanghai Artificial Intelligence Laboratory, Shanghai, 200232 China

**Keywords:** Breast cancer, Cancer imaging, Data publication and archiving, Medical imaging, Image processing

## Abstract

The success of training computer-vision models heavily relies on the support of large-scale, real-world images with annotations. Yet such an annotation-ready dataset is difficult to curate in pathology due to the privacy protection and excessive annotation burden. To aid in computational pathology, synthetic data generation, curation, and annotation present a cost-effective means to quickly enable data diversity that is required to boost model performance at different stages. In this study, we introduce a large-scale synthetic pathological image dataset paired with the annotation for nuclei semantic segmentation, termed as Synthetic Nuclei and annOtation Wizard (SNOW). The proposed SNOW is developed via a standardized workflow by applying the off-the-shelf image generator and nuclei annotator. The dataset contains overall 20k image tiles and 1,448,522 annotated nuclei with the CC-BY license. We show that SNOW can be used in both supervised and semi-supervised training scenarios. Extensive results suggest that synthetic-data-trained models are competitive under a variety of model training settings, expanding the scope of better using synthetic images for enhancing downstream data-driven clinical tasks.

## Background & Summary

Breast cancer is the most prevalent type of cancer in women with 2.3 million new cases annually^1^. Whole slide images (WSIs) offer an efficient tool to assess the disease visual status ranging from staging, metastasis, to prognosis^2,3^. Especially high-resolution WSI can capture diverse tissue appearance and cell morphology to inform clinical decision making. To advance diagnosis of breast cancer, nuclei identification plays a key role because its characteristics are strongly associated with patient outcomes^4^. For instance, aggressive breast cancers often include vesicular nuclei and prominent nucleoli^5^. Building a quantitative pipeline of nuclei analysis is thus crucial for understanding cancer microenvironment. Growing deep-learning approaches^3,4^ are developed to automate WSI-based nuclei segmentation, however, the model performance is contingent on the quality and quantity of real-world training samples.

Data set preparation and construction is becoming essential to deep-learning applications in pathology, where the present standard is to train model networks on curated real-world datasets^6^, and fine-tune the model until convergence^7–9^. Yet, pathological data curation faces daunting challenges in the healthcare system due to privacy protection and excessive annotation burden^10,11^. Annotating tissue characteristics from a complex WSI can take on average 1.8 hours^12^, thus scaling human-level WSI annotation is increasingly challenging. To date, real-world breast cancer-related nuclei data sets (Table 1) cover a limited range of samples (e.g., from 50 to 7,901) from BreCaHAD^13^, PanNuke^14,15^, and TNBC^16^. These cohorts still lack the size necessary to advance deep learning. Instead, synthetic data generation presents a cost-effective means to enable data diversity, holding the promise to boost model training for downstream tasks at different stages^17^.

**Table 1:** Summary of the datasets used in our experiments.

Dataset name	BreCaHAD^13^	PanNuke^14,15^	TNBC^16^	SNOW (Ours)
Dataset role	Image generation	Nuclei annotation	Model evaluation	Model training
Dataset type	Real-world	Real-world	Real-world	Synthetic
Number of tiles	176	7,901	50	**20**,**000**
Number of annotated nuclei	23,549	216,400	4,022	**1**,**448**,**522**
Size of tiles	1,360 × 1,024	256 × 256	512 × 512	512 × 512
Magnification	40×	40×	40×	40×
Tissue(s)	Breast	Breast and other tissues	Breast	Synthetic breast

Generative adversarial networks (GANs) have been a key computational tool for producing high-quality synthetic images^18–24^. However, GAN-based approaches are unable to directly generate the corresponding nuclei segmentation outcomes (i.e., nuclei masks). To address this limitation, in cancer pathology, researchers apply mask-defined approaches^25–29^ to generate synthetic images for improving cell semantic segmentation (i.e., the task of classifying each image pixel into either background or nuclei). These approaches usually begin with proposing predefined masks to locate nuclei positions. Examples include random-, distributional-generated masks^29^ and human-annotated masks^27–29^. Under such a mask-constrained setting, the use of GANs is prone to generate only shape-predefined nuclei samples. For instance, a predefined mask focusing on capturing fully-separated nuclei is unlikely to include the clumped nuclei, which is commonly observed in real-world tissue images. As a result, this predefined setting becomes a hurdle to training robust models for segmenting challenging cells (supplementary Table S1 and Table S2).

**Fig. 1:**
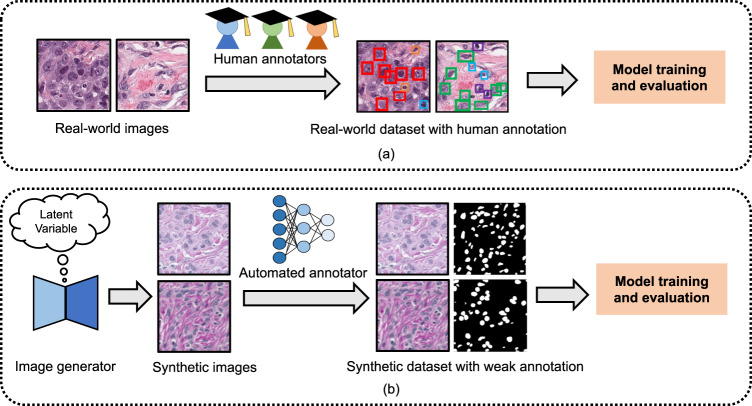
Comparison of data and model training pipeline. (**a**) Conventional model training pipeline. Conventional model training heavily relies on real-world images and laborious human-expert annotations. (**b**) Synthetic-data-enabled model training pipeline. By comparison, we focus on a pure synthetic image generation and annotation. We use the off-the-shelf GAN model as an image generator to yield informative synthetic images and then annotate the generated images by the weakly trained annotator.

To respond the lack of sizeable image data sets, we introduce a data set based on synthetic data generation and annotation from standardised, scalable, and reproducible perspectives. We propose the synthetic nuclei and annotation wizard (**SNOW**) (Fig. 1), which allows diverse synthetic data generation of breast cancer nuclei without any prior mask constraints. Our major focus is placed on the synthetic dataset curation, remarkably differing from studies seeking the parameter optimization of models^25–29^ or generating real-world data set^30^. Specifically, we address data-centric questions regarding the synthetic data set generation, evaluation, and quality in our study. First, can we generate informative synthetic samples via the use of limited public training data with minimal human effort? Second, which type of training strategy is desired when using large-scale synthetic images for nuclei semantic segmentation? Finally, can synthetic images alone serve as surrogate of real-world data sets for nuclei semantic segmentation?

**Fig. 2:**
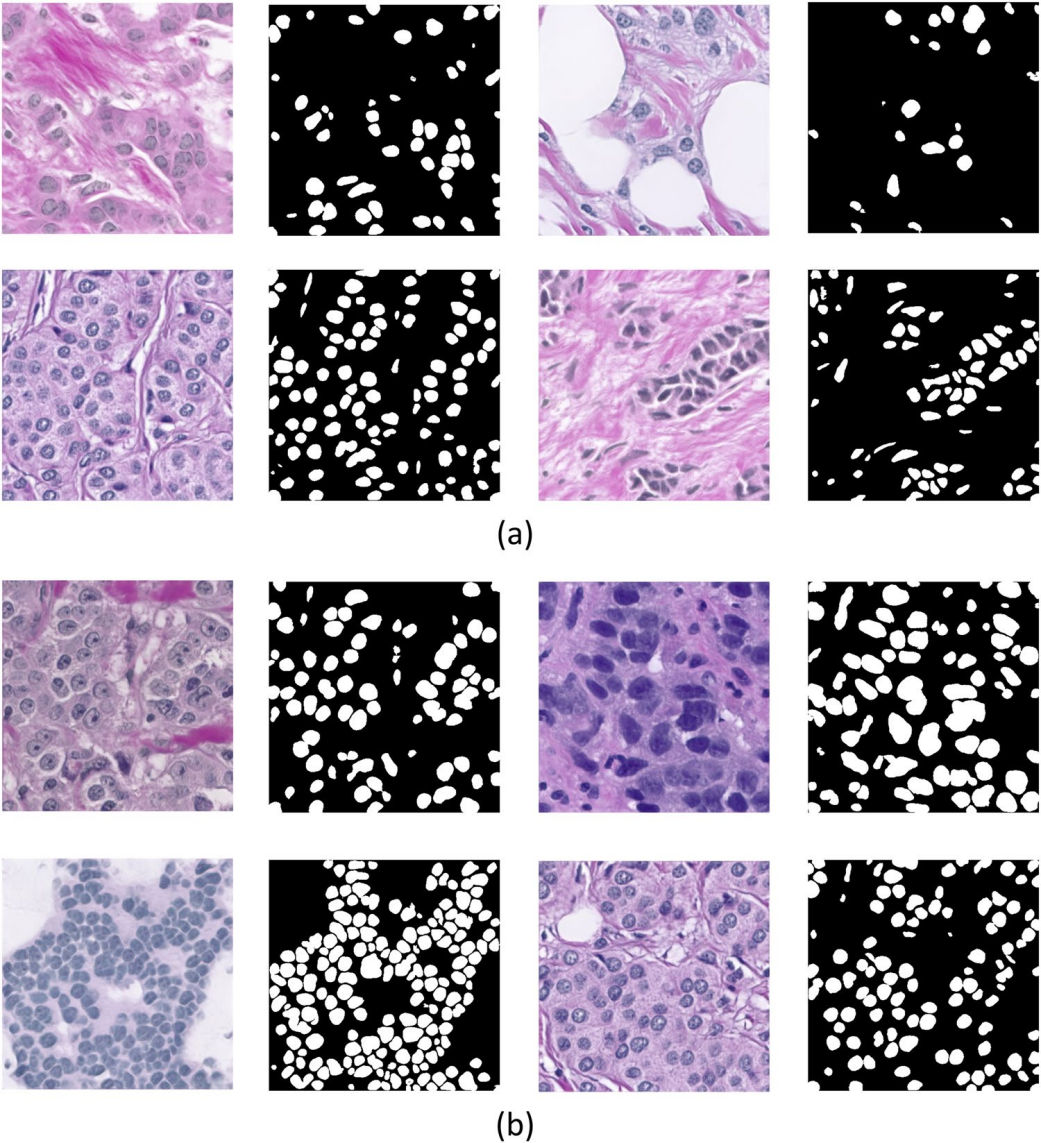
Synthetic image examples and annotations of SNOW data set. (**a**) Common nuclei examples (e.g., major nuclei are separated). (**b**) Challenging nuclei examples (e.g., closely adjacent or clumped nuclei).

SNOW data set (Fig. 2) opens up perspectives on the utility of meaningful synthetic data in pathological image assessment. This simple yet useful pipeline, together with quality verification on image contents, has demonstrated that training models from synthetic images alone can achieve competitive performance for nuclei semantic segmentation. Together, we expect SNOW to be a key resource for deep-learning nuclei segmentation, expanding the landscape of data curation in computational pathology. To extend, SNOW can potentially facilitate a variety of downstream image-based tasks, such as tumor staging, prognosis, and molecular analysis^31–35^. SNOW could also facilitate breast-cancer-specific pretraining and finetuning tasks. SNOW provides an alternative set of samples for the model training for the downstream task (e.g., only about 2,351 breast cancer data in PanNuke^14,15^ data set, which is 11% of SNOW data set). In addition, SNOW dataset could be used as the source data for training adversarial attack detection models to recognize malicious attacks in medical image analysis. Despite showing appealing characteristics of SNOW, we limit our focus on breast carcinoma in this report. A broader range of exploration on multiple cancers is the natural next step. Our study is built upon the power of off-the-shelf models with a proven utility on pathological images^6,36^, while diffusion models^37^ are not considered due to their high computational costs (e.g., about two times more than off-the-shelf models). To sum up, the release of SNOW makes an important step towards curating synthetic data sets compared to the current machine-training workflows using real-world pathological data (Fig. 1).

## Methods

### Pipeline overview

**Fig. 3:**
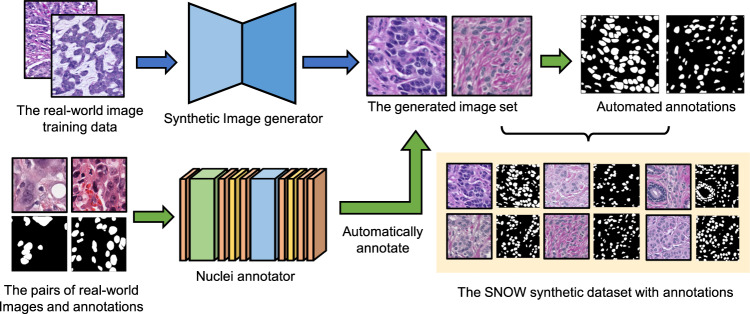
Overview of SNOW dataset pipeline. The pipeline contains a joint workflow of synthetic image generator (SIG) and nuclei annotator (NA). First, the real-world image training data include the high-resolution images from breast cancer histopathological annotation and diagnosis dataset (BreCaHAD)^13^. So we can train the synthetic image generator from scratch to generate synthetic breast tissue images from StyleGAN2. Next, PanNuke dataset^14,15^ provides pairs of image and annotation to weakly train the nuclei annotator to generate the needed annotation (e.g., nuclei mask) for synthetic images. The blue arrows represent the workflow of the synthetic image generation and the green arrows denote the workflow of nuclei annotation.

Figure 3 illustrates the major workflow of synthetic data set generation, including the synthetic image generator (SIG) and nuclei annotator (NA) for pathological images of breast carcinoma. The SIG produces vivid tiles based on training from a limited size of pathological training data (e.g. BreCaHAD^13^). The NA is designed to generate weak nuclei annotation (i.e., automatic annotation without manual correction) without adding fine-tuning procedures of nuclei annotator. Instead of pretraining on a large-scale, out-domain dataset (e.g., ImageNet), SIG and NA here is trained from scratch using in-domain public datasets given their differential characteristics on image resolution and annotation availability (Table 1). This design brings an efficient and reproducible means to generate paired synthetic image samples and annotations. SNOW also expands the current data scale of pathological nuclei analysis without adding human annotation efforts (e.g. overall 20k tiles and 1,448,522 annotated nuclei). Given this data, we perform extensive evaluation on measuring segmentation performance between using SNOW and real-world data sets.

### Synthetic image generator (SIG)

We define the synthetic image generator as an efficient tool to produce synthetic image tiles, including both tissues and nuclei. We offer two guiding principles about the design and purpose of the synthetic image generator, including image quality and data generation efficiency.

Synthetic image quality is evaluated by measuring the similarity between synthetic data to real-world data, including the nuclei shape and tissue morphology. Further, the nuclei statistics, such as the number of nuclei in each synthetic image, also reflect the quality of synthetic image. To satisfy these criteria, we choose StyleGAN2^36^ as our SIG because of its strong image generation ability. The StyleGAN2 generator maps a latent code *z* ∈ *Z* drawn from a normal distribution to a realistic image. Latent code z is first mapped to an intermediate latent code *w* ∈ *W* by a mapping function. Then, w is transformed to k vectors which are injected as style information into style blocks. To ensure the good image quality, StyleGAN2 utilized the perceptual path length (PPL) regularization^38^, which is used for estimating the quality of latent space interpolations and correlating with consistency and stability of shapes. Further, StyleGAN2 applies a truncation operation^38^ to add more diversity for image generation by avoiding the generated image converging to the ‘mean’ representation of the training dataset. These components make StyleGAN2 to produce the required synthetic images in our task.

To ensure the quality of image generation from StyleGAN2, data generation efficiency is a crucial factor in the domain of pathological image analysis. Considering a small-scale training data set, the discriminator of StyleGAN2 can overfit training samples, where the feedback to the generator of StyleGAN2 becomes less meaningful and training starts to diverge^39^. Standard data augmentation is helpful to alleviate the overfitting^39^, yet noisy augmentation inputs must be well handled. To address these issues, we use an adaptive discriminator augmentation (ADA) scheme in our SIG without changing the loss function or network architecture of StyleGAN2. The ADA scheme^39^ controls the augmentation strength by using a threshold p ∈ [0, 1] dynamically based on the degree of potential overfitting. The augmentation is applied with probability p or skipped with probability 1 - p). Inspired by RandAugment^40^, eighteen diverse transformations are included in the augmentation. The ADA scheme adjusts theshold p by evaluating the relevance between the StyleGAN2’s output on the original training samples and the generated images. Further, ADA evaluates the overfitting degree by estimating the portion of the training samples to get positive discriminator outputs. Without a large-scale out-domain pretraining (e.g., ImageNet), the use of ADA is proven to be helpful in a data-limited training environment^39^.

### Nuclei annotator (NA)

We define the nuclei annotator (NA) as a label-efficient expert to automatically accomplish nuclei annotation in synthetic images. Instead of pursuing fine-grained annotations, NA module only produces a set of weak nuclei annotations that perform reasonably good to train segmentation models without adding human correction. Currently, the routine workflow of nuclei semantic segmentation heavily depends on human annotation, which explains the restricted scale of nuclei dataset in the literature^13–15^. As opposed to using human annotation to scale up, we initialize NA module by leveraging public datasets with prior human knowledge of nuclei inputs. We selected HoVer-Net^6^ as the nuclei annotator in our study because of its reliable performance in multiple baselines of segmenting challenging nuclei examples^6,15^. In HoVer-Net, the feature extraction component is inspired by a pre-activated residual network with 50 layers (Preact-ResNet50)^41^. Compared to the standard Preact-ResNet50, HoVer-Net reduces the total down-sampling factor from 32 to 8 by using a stride of 1 in the first convolution and removing the subsequent maxpooling operation. These modifications reduce immediate loss of information and ensure the quality of segmentation^6^. HoVer-Net utilizes the nearest neighbour up-sampling via distinct functional branches, including nuclei pixel (NP) and HoVer branch. The NP branch predicts pixel membership to the nuclei or background, while the HoVer branch predicts the horizontal and vertical distances of nuclear pixels to their centres of mass^6^.

### Segmentation model training strategy

For supervised training scheme, we use all of the available images and the corresponding annotations for model training. The model *θ* is trained on the training set ${D}_{train}=\left\{\left({x}_{train}^{1},{y}_{train}^{1}\right),\left({x}_{train}^{2},{y}_{train}^{2}\right),...,\left({x}_{train}^{n},{y}_{train}^{n}\right)\right\}$. We optimize the model by minimizing the sum of binary cross-entropy *l*_*BCE*_ and dice loss *l*_*DICE*_. Further, we select the best model θ_*_ for model evaluation from the validation set, where n is the number of the training set.

1 $$L=\frac{1}{n}\mathop{\sum }\limits_{i=1}^{n}\left({l}_{BCE}\left({y}_{train}^{i},{\theta }_{\ast }\left({x}_{train}^{i}\right)\right)+{l}_{DICE}\left({y}_{train}^{i},{\theta }_{\ast }\left({x}_{train}^{i}\right)\right)\right)$$

For semi-supervised training scheme, we utilize a self-training strategy to enable a better exploration of the in-depth information hidden in the images. Inspired by^42^, we use the mix of labeled and unlabeled data to train the model. First, we train a teacher model *θ*^*teacher*^ on the labeled training set ${D}_{teacher}=\left\{\left({x}_{teacher}^{1},{y}_{teacher}^{1}\right),\left({x}_{teacher}^{2},{y}_{teacher}^{2}\right),...,\left({x}_{teacher}^{m},{y}_{teacher}^{m}\right)\right\}$ by minimizing the sum of binary cross-entropy *l*_*BCE*_ and dice loss *l*_*DICE*_. Moreover, we select the best teacher model ${\theta }_{\ast }^{teacher}$ by the labeled validation set.

2 $${L}_{labeled}=\frac{1}{m}\mathop{\sum }\limits_{i=1}^{m}\left({l}_{BCE}\left({y}_{teacher}^{i},{\theta }^{teacher}\left({x}_{teacher}^{i}\right)\right)+{l}_{DICE}\left({y}_{teacher}^{i},{\theta }^{teacher}\left({x}_{teacher}^{i}\right)\right)\right)$$

Where m is the number of labeled training data.

Then, we use the selected teacher model ${\theta }_{\ast }^{teacher}$ to generate soft pseudo annotations ${y}_{student}^{i}$ for unlabeled data ${D}_{student}=\left\{{x}_{student}^{1},{x}_{student}^{2},...,{x}_{student}^{m}\right\}$, where m is the number of unlabeled data.

3 $${y}_{student}^{i}={\theta }_{\ast }^{teacher}\left({x}_{student}^{i}\right)$$

We then train a student model *θ*^*student*^ on the labeled and pseudo-labeled data to minimize the combined loss. The student model has a same architecture and size as the teacher model.

4 $${L}_{unlabeled}=\frac{1}{m}\mathop{\sum }\limits_{i=1}^{m}\left({l}_{BCE}\left({y}_{student}^{i},{\theta }^{student}\left({x}_{student}^{i}\right)\right)+{l}_{DICE}\left({y}_{student}^{i},{\theta }^{student}\left({x}_{student}^{i}\right)\right)\right)$$

5 $${L}_{student}={L}_{labeled}+{L}_{unlabeled}$$

Further, the best student model is selected by measuring the performance from validation set. The selected student model ${\theta }_{\ast }^{student}$ will work as a new teacher model and generate new pseudo labels. We iteratively repeat the previous steps by using the best student model as the new teacher model to update the pseudo label, and training the student model until convergence.

## Data Records

**Fig. 4:**
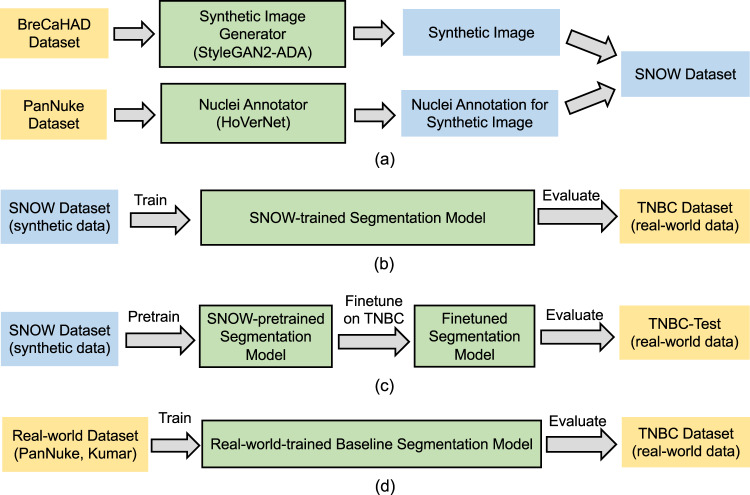
Evaluation workflow and dataset usage. (**a**) Synthetic dataset generation pipeline. In (**a**), we show the entire process of generating SNOW examples. (**b**) SNOW-trained model training and evaluation. In (**b**), we show the pipeline of measuring the performance of SNOW by training a segmentation model on SNOW and testing on the independent TNBC dataset. (**c**) Joint training on SNOW and real-world data set and evaluation. In (**c**), we show the workflow of assessing data set utility by using SNOW for in-domain pretraining and using TNBC training data for model finetuning. The finetuned model performance is then evaluated on the TNBC testing set. (**d**) Baseline model training and evaluation. In (**d**), we show the baseline comparisons by training on real-world datasets and testing on the TNBC as as well. The yellow and blue color boxes indicate the real-world and synthetic dataset respectively. The green color boxes represent the trained models accordingly.

The creation of our synthetic dataset is associated with two real-world data sets. Table 1 summarizes several public real-world data sets related to nuclei segmentation and the proposed SNOW data set (as illustrated in Fig. 4a). Due to the high-resolution detailed view of tissues (1,360 × 1,024), we use BreCaHAD to ensure the training performance of synthetic image generator. BreCaHAD data set^13^ includes 162 breast tissue tiles with point location for each nuclei. As BreCaHAD data set does not provide the semantic segmentation mask of entire nuclei (e.g., only point annotation), we utilize PanNuke data set^14,15^ for training nuclei annotator, which contains both images and corresponding annotation masks (e.g., nuclei is foreground and others are background). To date, PanNuke data set^14,15^ is the largest nuclei segmentation data set (tiles n = 7,901). Under the proposed pipeline, SNOW produces 20k synthetic breast cancer tissue patches paired with nuclei shape annotations.

### SNOW dataset

All data records are included in Zenodo (10.5281/zenodo.6633721^43^) and GitHub (https://github.com/Cassie07/SNOW-Dataset).

- **Image folder** All synthetic images are in SNOW_Image.zip folder.
- **Mask folders** Due to the large size of the nuclei segmentation mask, we split all masks into four folders (e.g., mask_0_5000.zip, mask_5001_10k.zip, mask_10k_15k.zip, and mask_15k_20k.zip). In each zip folder, we include three sub-folders: json, mat, and overlay. We only use mat folders to save nuclei segmentation mask for each synthetic image.
- **Datasheets for Datasets** To help users understand details of the dataset and possible extensive use, we prepared datasheets^44^ for our data set. The detailed responses are related to the following factors, including study motivation, composition, collection process, preprocessing/cleaning/labeling information, distributions, and data maintenance.

## Technical Validation

### Data set experimental settings and details

We apply nuclei semantic segmentation to evaluate the performance of the proposed synthetic dataset. To rigorously avoid data confusion, we did not use additional data augmentation during the training of segmentation models. Our major focus here is placed on diagnosing data utility, which differs from the majority of studies towards tuning model parameters for optimizing segmentation performance. In our experiment, we consider standard ResNet34^41^, DenseNet^45^ and Xception^46^ as the encoders in UNet^47^ architecture, which are widely used in medical image segmentation. After model training (as detailed below), we evaluate the segmentation performance on the independent, real-world dataset of Triple Negative Breast Cancer (TNBC) data set^16^. For all experiments in our study, the batch size is 64, the optimizer is Adam^48^, and the learning rate is 1e-4. We used four Tesla V100 SXM2 GPUs for our experiments. To evaluate nuclei semantic segmentation performance, we use the DICE score, Jaccard index (also known as Intersection-Over-Union (IoU)), and average hausdorff distance (aHD).

Regarding the synthetic image generator, we use the model of BreCaHAD-trained StyleGAN2-ADA. The model weights are released by https://github.com/NVlabs/stylegan2-ada-pytorch. The model weights are obtained by training StyleGAN2-ADA from scratch on BreCaHAD dataset. The batch size is 64, the optimizer is Adam, and the learning rate is 2.5e-3. Each image of BreCaHAD is cropped into patches with the resolution of 512 × 512 for model training. We choose BreCaHAD as a choice for generator input because of its key focus on nuclei assessment, where each image patch includes reasonable amounts of nuclei (e.g., about 145 nuclei per patch on average) for model training. This is in contrast with The Cancer Genome Atlas (TCGA) database^49^ containing WSIs without selection and annotation of nuclei, stromal cells, lymphocytes, and other tissue contents. For the design of image annotator, we use the PanNuke-trained HoVerNet model. The model weights are obtained from https://github.com/vqdang/hover_net. During the training process, the batch size is 8, the optimizer is Adam, and an initial learning rate of 1e-4 and then reduced it to 1e-5 after 25 epochs.

### Data set evaluation

Regarding data set evaluation, we consider two common training settings including supervised and semi-supervised training of segmentation models. Under each training strategy, we trained segmentation models on two types of data sets respectively, including the *real-world* data set (Fig. 4d) and the proposed *synthetic* data set (Fig. 4b). Note that for each experiment in Tables 2–4 and ablation studies, it is based on a strict separation of these two types of data sets without overlap, allowing us to assess the segmentation performance gained from the real-world data set and synthetic data set. Next, we detail two model training settings as below.

**Table 2:** (Supervised learning) The comparison of nuclei semantic segmentation performance on TNBC dataset. We trained the models on the real-world dataset and synthetic SNOW dataset separately to compare the performance difference derived from datasets. We use ImageNet-pretrained encoder in segmentation models. The results of first three rows were reported from^6^.

Number	Training Dataset Type	Dataset size	Segmentation model		DICE ↑ (%)	IoU ↑ (%)	aHD ↓
			Encoder	Architecture			
1	Real	30	—	UNet^47^	68.10	—	—
2	Real	30	—	DIST^16^	71.90	—	—
3	Real	30	—	HoVer-Net^6^	74.90	—	—
4	Real	7,901	ResNet34	UNet	76.77 ± 1.76	64.90 ± 1.37	8.04 ± 0.49
5	Real	7,901	DenseNet121	UNet	79.08 ± 0.92	68.45 ± 2.18	7.46 ± 0.22
6	Real	7,901	Xception	UNet	79.74 ± 0.36	67.05 ± 0.48	7.31 ± 0.04
7	Synthetic	20k	ResNet34	UNet	**80**.**25** ± **0**.**12**	**67**.**92** ± **0**.**20**	7.20 ± 0.02
8	Synthetic	20k	DenseNet121	UNet	79.90 ± 0.41	67.66 ± 0.62	**7**.**09** ± **0**.**03**
9	Synthetic	20k	Xception	UNet	80.08 ± 0.15	67.78 ± 0.19	7.12 ± 0.01

**Table 3:** (Semi-supervised learning) The comparison of nuclei semantic segmentation performance on TNBC dataset.

Number	Training Dataset Type	Dataset size	Segmentation model		DICE ↑ (%)	IoU ↑ (%)	aHD ↓
			Encoder	Architecture			
1	Real	7,901	ResNet34	UNet	75.45 ± 1.13	61.98 ± 1.04	7.84 ± 0.20
2	Real	7,901	DenseNet121	UNet	70.22 ± 0.90	56.47 ± 1.12	8.06 ± 0.40
3	Real	7,901	Xception	UNet	63.37 ± 2.13	50.31 ± 1.45	11.23 ± 0.18
4	Synthetic	20 k	ResNet34	UNet	**80**.**99** ± **0**.**25**	**68**.**70** ± **0**.**31**	**7**.**30** ± **0**.**04**
5	Synthetic	20 k	DenseNet121	UNet	79.15 ± 1.92	66.78 ± 2.17	7.37 ± 0.19
6	Synthetic	20 k	Xception	UNet	78.83 ± 0.14	65.96 ± 0.23	7.46 ± 0.06

We trained the models on the real-world dataset and synthetic SNOW dataset separately to compare the performance difference derived from datasets. We use ImageNet-pretrained encoder in segmentation models.

**Table 4:** Effect of pretrained encoder.

Dataset type	Dataset size	Encoder Pretrain	Training Mode	Annotation Ratio(%)	DICE ↑ (%)	IoU ↑ (%)	aHD ↓
Real	7,901	×	Supervised	100	73.26 ± 1.04	60.49 ± 1.08	8.29 ± 0.31
Real	7,901	×	Semi-supervised	50	69.74 ± 1.96	55.19 ± 1.85	8.95 ± 0.49
Synthetic	7,901	×	Supervised	100	76.17 ± 0.76	62.22 ± 0.94	7.53 ± 0.94
Synthetic	7,901	×	Semi-supervised	50	77.27 ± 1.08	63.49 ± 1.46	7.88 ± 0.09
Synthetic	20k	×	Supervised	100	77.16 ± 0.76	63.89 ± 0.93	**7**.**38** ± **0**.**01**
Synthetic	20k	×	Semi-supervised	50	**77**.**72** ± **0**.**47**	**64**.**15** ± **0**.**60**	7.84 ± 0.13

In this experiment, we focus on using the ResNet34 as the encoder of UNet segmentation architecture and training the segmentation model from scratch.

For supervised training (Table 2), we randomly split the used data set into the training set and the validation set (e.g., the split ratio is 95%, 5%). For the semi-supervised training (Table 3), we randomly split the data set into three sets, including the labeled teacher model training set, the labeled validation set, and the unlabeled set (e.g., the split ratio is 45%, 5%, and 50%). Thus the annotation ratio of the entire data set is 0.5. To prepare the unlabeled set, we use image samples only (i.e., no mask annotation is included). Next, we train the teacher model on the labeled teacher training set and select the best model by evaluating the model performance on the validation set. The best teacher model was used to generate the soft pseudo-label for the unlabeled set and worked as the initialized student model. Then, we trained the student model and selected the best student model as a new teacher model to update the unlabeled set annotation. Further, the best student model became the initialized model in the next iteration. After five iterations, we finally used the best student model for model evaluation. The number of epoch for teacher model and student model training is 100 and 15 respectively.

Table 2 shows that synthetic-data-trained models are competitive under the supervised training setting, outperforming state-of-the-art findings trained on real-world datasets. In the meantime, increasing the number of real-world samples (row 4–6) can improve results on the real-world data setting (row 1–3). It is no doubt that such a performance gain is based on the use of more real-world samples with an extra cost of human annotation. By comparison, when seeing results from synthetic data, models trained on SNOW dataset (row 7–9) are appealing to work as a cost-effective substitution for the real-world dataset (e.g., the DICE of synthetic-data trained model versus real-world data trained model is 80.25 ± 0.12 versus 76.77 ± 1.76 when using ResNet34 as the encoder of segmentation model)

^6^.

Table 3 reports the model nuclei semantic segmentation performance under the semi-supervised training setting. Similarly, we find that the segmentation models trained on SNOW dataset yield good performance. Applying ResNet34 encoder on SNOW achieves the highest score among all comparisons (Dice 80.99 ± 0.25). We reason this finding as a result of the machine-generated pseudo annotation that brings well generalization ability on unseen testing examples. By seeing the performance of real-world data using human-annotated masks, the results dropped considerably (Table 3, row 1–3) when comparing with their corresponding results via supervised learning (Table 2, row 4–6). This is reasonable given the smaller size of training data with human-provided annotation in semi-supervised learning. Notably, SNOW dataset provides a larger scale of training data that leads to the performance stability. To further validate the data set and reflect the benefits of SNOW for segmenting clumped nuclei, we extend the data set evaluation to the nuclei instance segmentation and achieve a similar finding (supplementary Table S3 and Table S4).

**Fig. 5:**
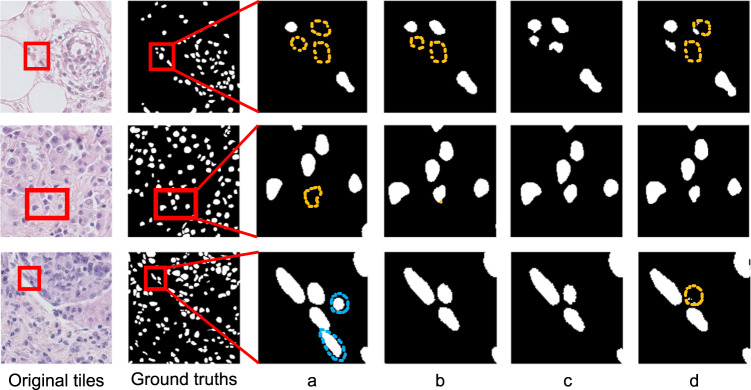
Visualization of segmentation performance on TNBC dataset. The first and second columns are original tiles and the associated ground truth. Columns a to d offer the zoom-in view of segmentation outputs that generated by the model (e.g., ResNet34 as encoder of the UNet architecture) with the best performance. The column a shows the segmentation performance when the model trained on the real-world dataset (e.g., PanNuke) under the supervised learning setting (e.g., in Table 2). The column b shows the segmentation performance of model trained on SNOW under the supervised learning setting (e.g., in Table 2). The column c shows the segmentation performance of model trained on SNOW under the semi-supervised learning setting (e.g., in Table 3). The column d shown the segmentation performance of model that are not pretrained on ImageNet (e.g., in Table 4). The yellow dotted regions represent missing nuclei and the blue ones denote redundant nuclei when comparing to the ground truth.

Figure 5 reveals the visual quality of segmentation details. We observe that the models trained on SNOW yield reasonable nuclei segmentation, while the models trained on the real-world data set may present missing (yellow) or abundant (blue) nuclei annotations. In particular, the self-training scheme (Fig. 5, column c) together with the SNOW data set achieves the improved quality of segmentation by yielding nuclei boundaries without missing or abundant segmentation outcomes. As shown in Fig. 2b,these examples reveal the possibility to identify challenging nuclei (e.g., closely adjacent nuclei), which can offer augmented decision making of nuclei assessment as opposed to the models trained only from real-world data.

As seen in Table 4, we evaluate the effect of using large-scale out-domain pretained encoder on segmentation results (i.e., comparing results with (Tables 2, 3) or without (Table 4) pretaining on ImageNet). A key insight is that synthetic-data-trained models retain a high-level performance, while the model trained on the real-world dataset drops evidently without out-domain pre-training. Compared with the real-world dataset, we see that SNOW alone is informative to train the model while reducing the dependency of large-scale, out-domain pretraining.

### Data set utility

To assess the extensible utility of SNOW, we evaluate a joint segmentation performance using synthetic and real-world data (as shown in Fig. 4c). In detail, we use SNOW (e.g., 20k) as in-domain pretraining data rather than directly combining them with real-world data as done in conventional data augmentation^50,51^. We then finetune the SNOW-trained model on the real-world in-domain TNBC data (70% as training set and 10% as validation set). Finally, the finetuned model will be tested on the remained real-world data TNBC (20%). In this pipeline, the in-domain pretraining is under either supervised and semi-supervised training strategy, while the finetuning is under supervised training mode. We choose a full-model weights trainable finetuning to update the model weights on TNBC training set. Under the above setting, we also report baseline results regarding whether or not using out-domain real-world data for model encoder initialization.

Table 5 shows that using SNOW provides benefits under several data scenarios. First, using SNOW as model training data can boost nuclei segmentation performance. In particular, we found that without using out-domain encoder pretrain (row 1–5), the segmentation model shows a good performance by using SNOW for in-domain pretraining (e.g., row 4 versus row 1, Dice 79.15 ± 0.39 versus 74.22 ± 0.82). Further, in-domain finetuning via a small size of real-world data set (e.g., 40 nuclei patches and associated masks) enhances segmentation performance (e.g., row 3, Dice 81.08 ± 0.32). Second, SNOW brings pretraining benefits when jointly cooperating with in-domain finetuning via a small amount (e.g., 40 nuclei patches) of real-world data. Compared with results from out-domain pretraining (row 6–10), we found that using SNOW as in-domain pretraining provides a comparable performance without finetuning (e.g., row 4 versus row 9, Dice 79.15 ± 0.39 versus 79.95 ± 0.52). Critically, we recognize that SNOW could serve as a bridge between natural and medical image domain, extending us to better leverage models that are initialized by large-scale out-domain data. For instance, with out-domain pretraining on large-scale natural image set, SNOW consistently boosts the segmentation performance on medical-domain data (e.g., row 10 versus row 6, Dice 82.42 ± 0.43 versus 75.98 ± 0.88). Interestingly, SNOW becomes helpful to model initialization and model finetuning under the same medical-domain. With an in-domain finetuning, the result only using in-domain pretraining slightly outperforms using in-domain and out-domain pretraining together (e.g., row 3 versus row 8, Dice 81.08 ± 0.32 versus 80.91 ± 0.78).

**Table 5:** Synthetic data set utility evaluation.

Number	Encoder Pretrain (Out-domain)	Pretrain Data (In-domain)	Pretrain mode (In-domain)	Finetuning Data (In-domain)	DICE ↑ (%)	IoU ↑ (%)	aHD ↓
1	×	—	—	TNBC	74.22 ± 0.82	59.24 ± 0.98	8.02 ± 0.14
2	×	SNOW-20k	supervised	—	75.69 ± 0.87	62.94 ± 0.99	6.63 ± 0.03
3	×	SNOW-20k	supervised	TNBC	81.08 ± 0.32	68.95 ± 0.25	6.92 ± 0.03
4	×	SNOW-20k	semi-supervised	—	79.15 ± 0.39	66.16 ± 0.54	7.20 ± 0.15
5	×	SNOW-20k	semi-supervised	TNBC	80.16 ± 1.04	67.63 ± 1.04	6.91 ± 0.25
6	✓	—	—	TNBC	75.98 ± 0.88	62.27 ± 0.78	7.29 ± 0.14
7	✓	SNOW-20k	supervised	—	77.71 ± 0.51	65.96 ± 0.60	**6**.**50** ± **0**.**01**
8	✓	SNOW-20k	supervised	TNBC	80.91 ± 0.78	69.23 ± 092	7.02 ± 0.20
9	✓	SNOW-20k	semi-supervised	—	79.95 ± 0.52	67.35 ± 0.82	6.91 ± 0.07
10	✓	SNOW-20k	semi-supervised	TNBC	**82**.**42** ± **0**.**43**	**70**.**53** ± **0**.**59**	6.93 ± 0.06

We use ResNet34 as the encoder of the UNet segmentation model, which is either initialized by ImageNet pre-trained weights (e.g., out-domain pretraining) or trained from scratch. In the bellow table, “pretrain data (in-domain)” represents that we use the entire SNOW data set (e.g., 20k) for the segmentation model training. “Finetuning data (in-domain)” means that we use the TNBC training and validation set for the segmentation model finetuning. Finally, all the experiments are evaluated on the TNBC testing set.

### Data set quality verification

**Fig. 6:**
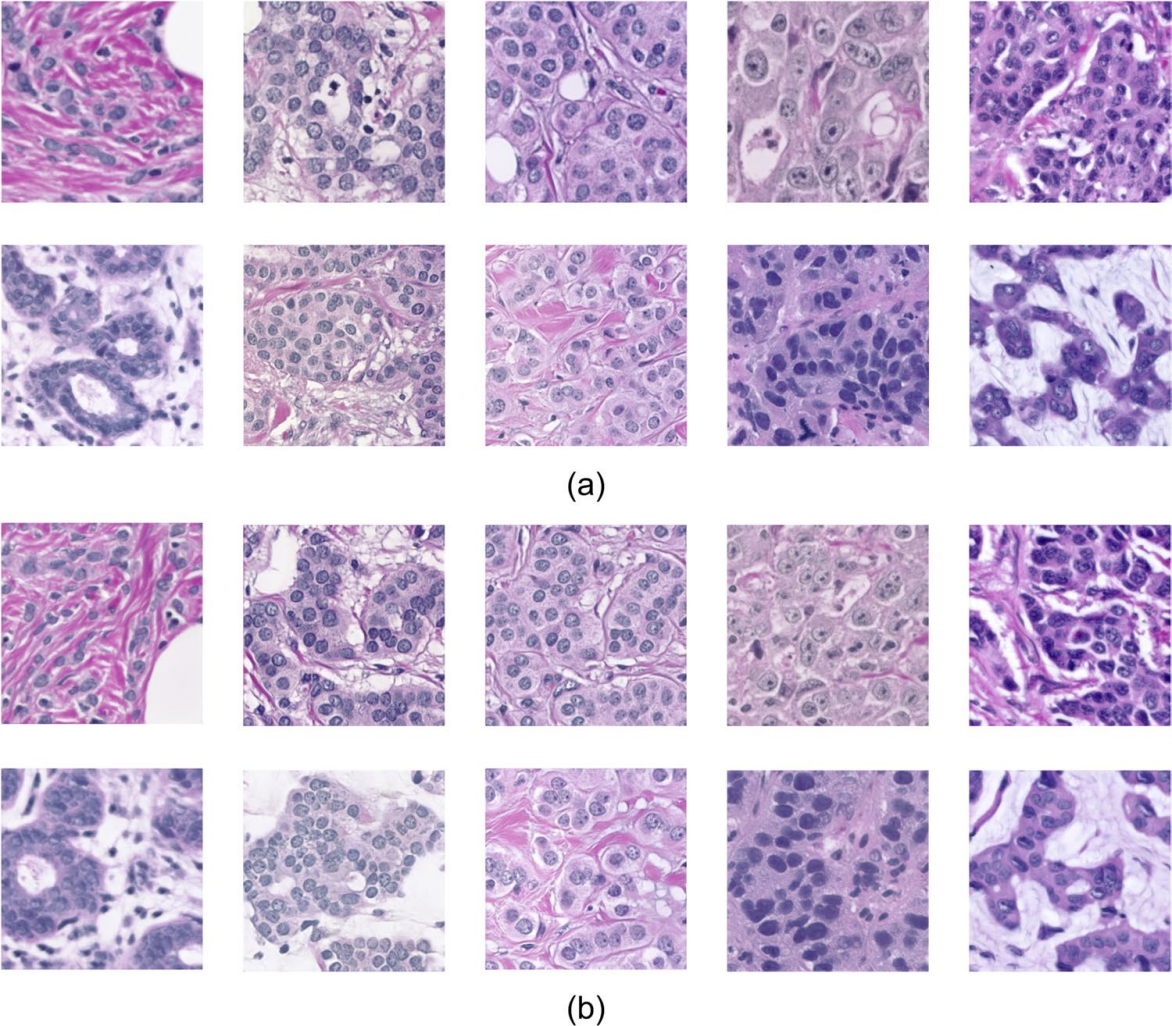
The comparison between the real-world and the synthetic images. (**a**) The real-world images for synthetic image generator training. In (**a**), we show the images from the BreCaHAD dataset. (**b**) The synthetic images. In (**b**), we show the representative synthetic images in SNOW dataset.

We evaluate the quality of both image contents and annotation performance of our synthetic data set. For measuring the quality of image contents, we use the numerical value of Fréchet inception distance (FID)^52^ to quantitatively analyze the quality of the synthetic images, which reflects the feature distance between the real-world and the generated image^53^. The average FID is 16.82 across the entire synthetic images. As shown in Fig. 6, the synthetic images present reasonable visual appearances as compared to the real-world dataset observed by human eyes.

We extend to assess the reliability performance of the nuclei annotator on the unseen data sets. We use the trained annotator to annotate multiple public nuclei segmentation data sets and comparing the results with the associated ground-truth annotations provided by human experts. First, we report the internal nuclei annotator capability by annotating PanNuke data set because the nuclei annotator is trained and evaluated by the same data set. Second, we assess the ability of nuclei annotator by evaluating on unseen data sets (e.g., TNBC^16^ and CoNSeP dataset^6^). In Table 6, it is remarkable that findings on the unseen external data sets are close to the values of the PanNuke dataset, reiterating the potential capability of annotator on unseen image examples.

**Table 6:** Nuclei annotator quality analysis. In this experiment, we use HoVerNet^6^ as nuclei annotator to annotate public datasets.

Datasets	Dice (%) ↑	IoU (%) ↑	aHD ↓
PanNuke^14,15^	84.72	73.21	6.13
TNBC^16^	81.61	69.83	7.01
CoNSeP^6^	81.81	69.74	10.94

### Ablation study analysis

**Fig. 7:**
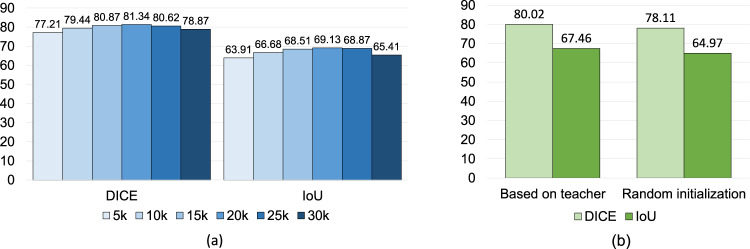
Ablation studies. (**a**) The sizes of synthetic dataset. In a, we evaluated the effect of the synthetic dataset sizes. (**b**) The initialization of student model. In b, we evaluated the effect of student model initialization in the semi-supervised training scheme.

In Fig. 7a, we generate different sizes of the synthetic dataset (e.g., 5k to 30k) to evaluate the size effect. We find that the performance on 20K outperforms other sizes of the dataset, while the model performance slightly decreases with a larger dataset size due to the potential added noise. Hence, we fix the dataset of 20k in size for major experiments considering the trade-off between computational efficiency and the performance. Further, we observe that with the similar quantity of the real-world and synthetic dataset, the performance of the synthetic dataset still achieves superior performance (e.g., The DICE score of the semantic segmentation model trained on synthetic versus real-world dataset is 77.21–79.44 versus 75.45). Interestingly, as shown in Fig. 7a and Table 3, even with less data (e.g., synthetic versus real-world is 5,000 versus 7,901), the synthetic-data trained model could achieve a better performance than the real-world trained model (e.g., 77.21 versus 75.45).

In Fig. 7b, we evaluate the model performance under the semi-supervised setting with different student model initializations. For all experiments in Fig. 7b, we used ResNet34 as segmentation model encoder with pretrained weights on ImageNet. We observe that initializing student model based on the trained teacher model resulted in a better segmentation performance than a random initialization. This finding on medical data is distinct from the observation in the self-training^42^ applied on the natural image dataset.

## Usage Notes

We use CC-BY license and complete attribution metadata.

## Supplementary information

Supplementary information

## Data Availability

All code is available on on Github https://github.com/Cassie07/SNOW-Dataset.
