# Supplementary material for: A Large-scale Synthetic Pathological Dataset for Deep Learning-enabled Segmentation of Breast Cancer
Source: Sci Data. 2023 Apr 21;10:231. doi: 10.1038/s41597-023-02125-y (PMC10121551; doi:10.1038/s41597-023-02125-y)
Supplement: Supplementary file 1 — Supplementary information [file 41597_2023_2125_MOESM1_ESM.pdf]

Supplementary Information for

A Large-scale Synthetic Pathological Dataset for Deep Learning-enabled Segmentation of Breast Cancer

Kexin Ding[1], Mu Zhou[2], He Wang[3], Olivier Gevaert[4], Dimitris Metaxas[5], Shaoting Zhang[6]*

1. Department of Computer Science, University of North Carolina at Charlotte, Charlotte, NC, 28262, United States
2. Sensebrain Research, San Jose, CA, 95131, United States
3. Department of Pathology, Yale University, New Haven, CT 06520, United States
4. Stanford Center for Biomedical Informatics Research, Department of Medicine and Biomedical Data Science, Stanford University, Stanford, CA, 94305, United States
5. Department of Computer Science, Rutgers University, New Brunswick, NJ, 08901, United States
6. Shanghai Artificial Intelligence Laboratory, Shanghai, 200232, China

Corresponding Author
* Shaoting Zhang (zhangshaoting@pjlab.org.cn)

Content

Comparison between SNOW and Shape-predefined Synthetic Data Set ……………2
Table S1 (Supervised learning) The comparison of segmentation performance on TNBC dataset. ………………………………………………………………………………………2
Table S2 (Semi-supervised learning) The comparison of segmentation performance on TNBC dataset. ………………………………………………………………………………………3

Data Set Evaluation Extension on Nuclei Instance Segmentation……………………3
Table S3 (Supervised learning) The comparison of nuclei instance segmentation performance on TNBC dataset. ………………………………………………………………………………4

Table S4 (Semi-supervised learning) The comparison of nuclei instance segmentation performance on TNBC dataset. ……………………………………………………………………4

Comparison between SNOW and Shape-predefined Synthetic Data Set

We compared the nuclei segmentation performance by training the model on our proposed SNOW data set (i.e., computer-annotated data set) and the shape-predefined nuclei data set, respectively. Generally, shape-predefined nuclei data set used the randomly-polynomial nuclei mask or the real-world nuclei mask together with the real-world image to generated synthetic image. Compared with a synthetic data generation pipeline, the shape-predefined nuclei data set generation is more likely to be a "domain adaptation" process in which the source domain is the predefined nuclei mask, and the target domain is the real-world pathological images. We utilized the pipeline in the previous study[1] that used a CycleGAN to generate the corresponding synthetic histopathological images for the shape-predefined nuclei masks.

We trained the nuclei segmentation models on both computer-annotated and shape-predefined data sets following the same setting in the main manuscript. The results are shown in Table S1 and Table S2 We observed that our computer-annotated data sets could achieve a better semantic segmentation performance than the shape-predefined data sets on both nuclei semantic segmentation and instance segmentation tasks under supervised and semi-supervised training schemes. We found that the shape-predefined data set generation highly depends on the quality of randomly-generated polynomial nuclei masks. Meanwhile, as observed, the nuclei in the synthetic images are potentially larger than the predefined nuclei mask, which likely leads to inaccurate model training and segmentation performance. The above issue has been avoided in the SNOW data set because we generated a synthetic image first and annotated the nuclei relying on the synthetic image. This process is closer to the nuclei annotation workflow as done by human experts.

Table S1. (Supervised learning) The comparison of segmentation performance on TNBC dataset. We trained the models on the shape-predefined synthetic dataset and the proposed SNOW dataset separately to compare the performance difference derived from datasets. We use ImageNet-pretrained encoder in segmentation models. The results of first three rows were reported from[2].

Annotation type	Dataset size	Segmentation model	DICE ↑ (%)	IoU ↑ (%)	aHD ↓	AJI ↑ (%)	PQ ↑ (%)
Shape-predefined annotation	20k	ResNet34-Unet	78.35	65.34	7.29	59.80	57.83
Shape-predefined annotation	20k	DenseNet121-Unet	78.12	65.49	7.15	59.91	58.42
Shape-predefined annotation	20k	Xception-Unet	77.54	64.27	7.42	59.42	54.28
Computer-annotated	20k	ResNet34-Unet	**80.25**	**67.92**	7.20	57.67	57.76
Computer-annotated	20k	DenseNet121-Unet	79.90	67.66	**7.09**	60.65	**59.72**

Computer-annotated	20k	Xception-Unet	80.08	67.78	7.12	**61.30**	59.14

Table S2. (Semi-supervised learning) The comparison of segmentation performance on TNBC dataset. We trained the models on the shape-predefined synthetic dataset and the proposed SNOW dataset separately to compare the performance difference derived from datasets. We use ImageNet-pretrained encoder in segmentation models.

Annotation type	Dataset size	Segmentation model	DICE ↑ (%)	IoU ↑ (%)	aHD ↓	AJI ↑ (%)	PQ ↑ (%)
Shape-predefined annotation	20k	ResNet34-Unet	76.46	62.78	8.25	57.10	54.27
Shape-predefined annotation	20k	DenseNet121-Unet	78.84	65.84	7.76	57.82	54.11
Shape-predefined annotation	20k	Xception-Unet	71.78	57.82	8.90	52.58	47.07
Computer-annotated	20k	ResNet34-Unet	**80.99**	**68.70**	**7.30**	**61.35**	**59.47**
Computer-annotated	20k	DenseNet121-Unet	79.15	66.78	7.37	60.67	58.61
Computer-annotated	20k	Xception-Unet	78.83	65.96	7.46	59.32	57.97

Data Set Evaluation Extension on Nuclei Instance Segmentation

To extensively evaluate the benefits of the SNOW data set to nuclei segmentation, we extend the data set evaluation task into nuclei instance segmentation and keep the same experiment settings as in the main manuscript. The nuclei instance segmentation task could reflect the model capability of segmenting clumped nuclei. Under the same model selection as in previous sections, we added a simple post-process method to convert the output (e.g., binary mask) of segmentation model to an instance segmentation mask. The simple post-process method mainly detects the connected-components to determine the nuclei instances and remove the small debris (e.g., the number of pixels in connected-components smaller than 10). To evaluate nuclei semantic segmentation performance, we use the Average Jaccard index (AJI)[3], Panoptic Quality (PQ)[2], the detection quality (DQ), and Segmentation quality (SQ) to evaluate nuclei instance segmentation performance. PQ is calculated by the DQ, which is the F1 score widely used to evaluate instance detection, and SQ, which reflects how close each correctly detected instance is to the matched ground truth.

We summarized the nuclei instance segmentation results in Table S3 and Table S4. Similar to the nuclei semantic segmentation, we found that the nuclei instance segmentation models trained on the proposed SNOW data set could yield a better performance under both supervised and semi-supervised model training settings. Significantly, the models trained on the SNOW data set always outperform the models trained on the real-world data set with a semi-supervised model training strategy. As shown in Table S3 and Table S4, similar to the nuclei semantic segmentation, we also recognized that the nuclei instance segmentation models trained on the proposed SNOW data set could yield a better performance under both supervised and semi-supervised model training. Even with a simple nuclei instance segmentation training post-processing, the models trained on the SNOW dataset could always outperform the models trained on the real-world dataset in the supervised and semi-supervised training schemes. For example, in Table S3, the SNOW-trained models could achieve about 2% AJI and PQ improvement over the Real-world-trained models. In Table S4, the SNOW-trained models could yield a significant improvement (e.g., about 7% AJI improvement and 5% PQ improvement), while the real-world-trained models could not perform well for nuclei instance segmentation tasks.

Table S3. (Supervised learning) The comparison of nuclei instance segmentation performance on TNBC dataset. We trained the models on the real-world dataset and synthetic SNOW dataset separately to compare the performance difference derived from datasets. We use ImageNet-pretrained encoder in segmentation models. The results of first three rows were reported from[2].

**Dataset type**	**Dataset size**	**Segmentation model**	**AJI ↑ (%)**	**DQ ↑ (%)**	**SQ ↑ (%)**	**PQ ↑ (%)**
Real	30	UNet	51.40	63.50	67.60	44.20
Real	30	DIST	52.30	54.90	71.40	40.40
Real	30	HoVer-Net	59.00	74.30	75.90	57.80
Real	7,901	ResNet34-Unet	57.67	74.00	77.76	57.76
Real	7,901	DenseNet121-Unet	59.31	74.72	78.31	58.69
Real	7,901	Xception-Unet	60.87	75.13	78.05	58.80
Synthetic	20k	ResNet34-Unet	61.16	**75.28**	78.66	59.45
Synthetic	20k	DenseNet121-Unet	60.65	75.22	**79.06**	**59.72**
Synthetic	20k	Xception-Unet	**61.30**	74.75	78.78	59.14

Table S4. (Semi-supervised learning) The comparison of nuclei instance segmentation performance on TNBC dataset. We trained the models on the real-world dataset and synthetic SNOW dataset separately to compare the performance difference derived from datasets. We use ImageNet-pretrained encoder in segmentation models.

Dataset type	Dataset size	Segmentation model	AJI ↑ (%)	DQ ↑ (%)	SQ ↑ (%)	PQ ↑ (%)
Real	7,901	ResNet34-Unet	54.72	70.45	75.36	54.21
Real	7,901	DenseNet121-Unet	48.57	62.91	73.07	48.04
Real	7,901	Xception-Unet	41.12	59.64	72.48	45.28
Synthetic	20k	ResNet34-Unet	**61.35**	**74.89**	**79.27**	**59.47**
Synthetic	20k	DenseNet121-Unet	60.67	73.90	79.12	58.61
Synthetic	20k	Xception-Unet	59.32	73.21	78.87	57.97

References

1. Mahmood, F. et al. Deep adversarial training for multi-organ nuclei segmentation in histopathology images. IEEE transactions on medical imaging 39, 3257–3267 (2019).

2. Graham, S. et al. Hover-net: Simultaneous segmentation and classification of nuclei in multi-tissue histology images. Med. Image Analysis 58, 101563 (2019).

3. Kumar, N. et al. A dataset and a technique for generalized nuclear segmentation for computational pathology. IEEE transactions on medical imaging 36, 1550–1560 (2017).
